# Shale Gas Nanofluid in the Curved Carbon Nanotube: A Molecular Dynamics Simulation Study

**DOI:** 10.1021/acsomega.4c03659

**Published:** 2024-07-05

**Authors:** Jiang Wang, Zhiling Li, Wenli Zhang

**Affiliations:** † College of Science, Guizhou Institute of Technology, Boshi Road, Dangwu Town, Gui’an New District, Guizhou 550025, China; ‡ School of Transportation Engineering, Guizhou Institute of Technology, Boshi Road, Dangwu Town, Gui’an New District, Guizhou 550025, China

## Abstract

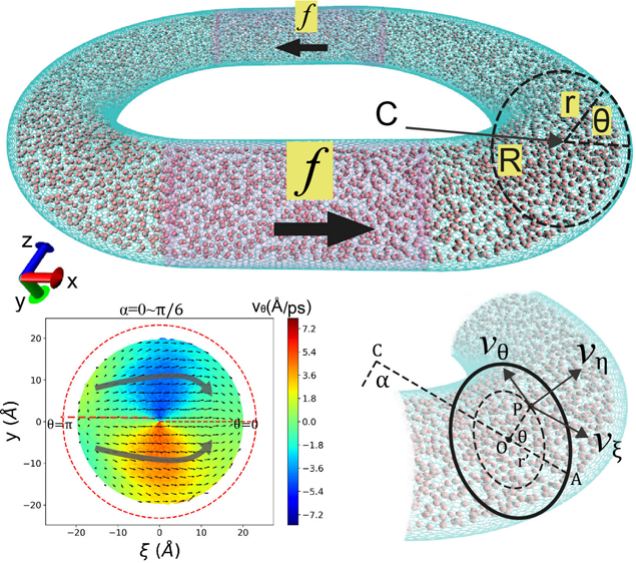

Curved nanochannels are prevalent in porous and tortuous materials, with shale matrices being a noteworthy example. The tortuosity of shale matrices significantly influences the behavior of shale gas, holding crucial implications for gas recovery engineering. In this study, we employ molecular dynamics simulation (MD) to investigate the impact of curvature and radius in tortuous nanochannel formed by a curved single-walled carbon nanotube (SWCNT) on the adsorption and transport properties of methane gas fluid. Our findings reveal that the inner half surface of the SWCNT, characterized by negative curvature, exhibits enhanced methane adsorption. Methane in straighter and narrower channels displays higher flow velocities, while wider channels exhibit higher flow flux. The nonzero flow velocity alters adsorption strength, causing the outer half to surpass the inner half. Tangent and vertical velocities of the flow are heterogeneously distributed in the channel, with the outer half having higher tangent velocities. Additionally, a vertical velocity pulse near the entrance induces turbulent vortex flow, slowing down the tangent flow velocity. This research contributes to a deeper understanding of shale gas properties in matrices with bent and curved channels, offering insights into nanofluids in carbon nanotubes and porous media featuring curved nanochannels.

## 1 Introduction

The increasing global demand for energy and fuels has propelled shale gas into prominence as a crucial energy resource. Its substantial storage capacity and relatively cleaner emissions compared to other fossil fuels make it a key player in the global energy landscape.^1−9^

Primarily composed of methane, shale gas is predominantly found in the organic porous structures of the shale matrix, commonly referred to as kerogen.^4,10−14^ Modern experimental techniques, including small-angle neutron scattering (SANS),^15,16^ mercury intrusion,^17,18^ atomic force microscopy (AFM),^19^ have been employed to reveal the porous and fractal nature of kerogen. With methods like scanning electron microscopy (SEM),^20−22^ nanopore structure could be directly observed in images. These studies indicate that nanopores are intricately connected by fractal nanochannels, with dimensions ranging from 1 to 100 nm.^15−20,23−25^ Notably, various shapes of nanochannel cross sections have been identified, including slit, rectangle, triangle, or circle.^26^

Recent experimental and numerical investigations have provided more comprehensive insights into the properties of nanopores within the shale matrix. Similar to other organic porous materials, the shale matrix exhibits tortuosity, characterized by curved and bent structures in nanochannels throughout.^21,27−30^ The tortuosity (τ) of the medium is typically defined as τ = *C*/*L*, where *L* represents the distance along the straight path connecting two points, and *C* is the arc length of the curved path along the nanochannel connecting the same points.^31,32^

Under high-temperature and high-pressure conditions deep underground, shale gas undergoes a transition into a supercritical fluid, exhibiting properties of both gases and liquids.^33,34^ In this state, methane molecules within shale gas display a pronounced tendency to be adsorbed onto the surface of nanopores. Simultaneously, methane can transport along nanochannels due to pressure gradients. A deeper understanding of these properties is crucial for optimizing the recovery of gas resources,^35−38^ and could also deepen our knowledge on other nanoflow in porous nano materials.^39,40^

However, exploring the dynamic properties of methane within such small-scale nanopores presents a considerable challenge.^41−46^ Molecular dynamics simulation (MD) has emerged as an effective tool, enabling access to the nanometer-scale dynamic behavior of methane in nanopores.^42,43,45,47−58^

MD has been employed to investigate diverse properties of shale gas in kerogen nanopores. This includes exploring adsorption and transport behaviors in nanochannels with different cross-sectional shapes,^26^ varying roughness of the wall surface,^59,60^ and nanochannels containing functional groups.^61,62^ Stickly layer also impact the transport of methane molecules,^63^ and carbon dioxide could enhance gas recovery.^64,65^ In our recent work, we delved into the adsorption and transport behavior of methane nanofluid in straight nanoslits, considering different π – π stacking configurations.^66^ However, the majority of existing research has primarily explored properties in straight nanochannels, overlooking the effects of curvature and tortuosity.

**Figure 1:**
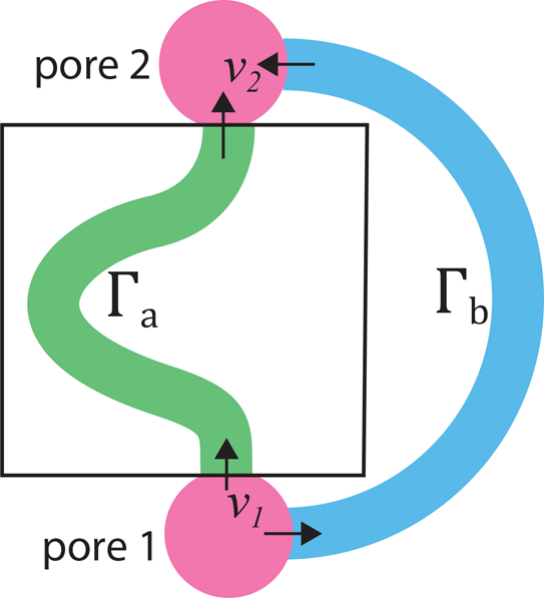
In the traditional simulation of nanofluids in a box with a periodic condition, the entrance and the exit of the Γ_*a*_ nanochannel (green) are connected to each other, result in the same entering and exiting velocity (*v⃗*_1_ = *v⃗*_2_). But in real situations, nanochannels connecting nanopore 1 and nanopore 2 could have different entering and exiting velocities, as shown in the blue channel Γ_*b*_, the angle between *v⃗*_1_ and *v⃗*_2_ is π, which cannot be simulated in a box with periodic boundary conditions.

In several recent studies, MD has been employed to elucidate methane flow behavior in tortuous channels. Zhang et al. utilized MD to simulate methane flow in a nanochannel with italic and turning angles, revealing that the italic angle enhances flow velocities.^67^ Ramírez et al. conducted simulations of methane fluid in nanotubes with tortuosity and roughness, observing that both roughness and curvature impact flux, with roughness playing a more prominent role. Additionally, nanotubes with sudden turnings were found to decelerate flow velocity.^68^ Notably, existing MD research on methane flow has, to the best of our knowledge, been conducted within boxes employing periodic boundary conditions. In such cases, fluid velocities at the entrance and exit are identical, which may differ from real-world scenarios, as illustrate in Figure 1.

Single-walled carbon nanotube (SWCNT), a long rod formed by rolling up a single-layer graphene sheet, possesses a high aspect ratio,^69^ has proven to be an idealized and straightforward model for investigating various nanofluids.^70−74^ Previous experimental and theoretical research has demonstrated that the smoothness of the SWCNT surface can enhance flow flux,^60,70,71^ with water molecule velocities reaching up to 10^3^ m/s.^75^ Additionally, studies by Zhao et al. have shown that graphene effectively represents kerogen, accurately capturing methane-kerogen interaction potentials.^76^

Leveraging these insights, in this research, we would utilize molecular dynamics simulation to investigate the adsorption and flow hehavior of methane molecules in toutuous nano channels. We devise a tortuous nanochannel under circular periodic conditions using a closed curved single-walled carbon nanotube (SWCNT). The circular cross-section of SWCNT is well aligned with previous findings suggesting its prevalence in organic shale matrices.^77,78^ Grounded in this model, our investigation will delve into the properties of methane adsorption, flux, and flow velocity within curved SWCNT.

This research is useful to advance our comprehension of shale gas properties within shale matrices featuring curved nanochannels. Furthermore, it has the potential to offer valuable insights into the behavior of other nanofluids in systems such as carbon nanotubes (CNTs) or intricate porous media.

## 2 Methods

### 2.1 The Construction of SWCNT

**Figure 2:**
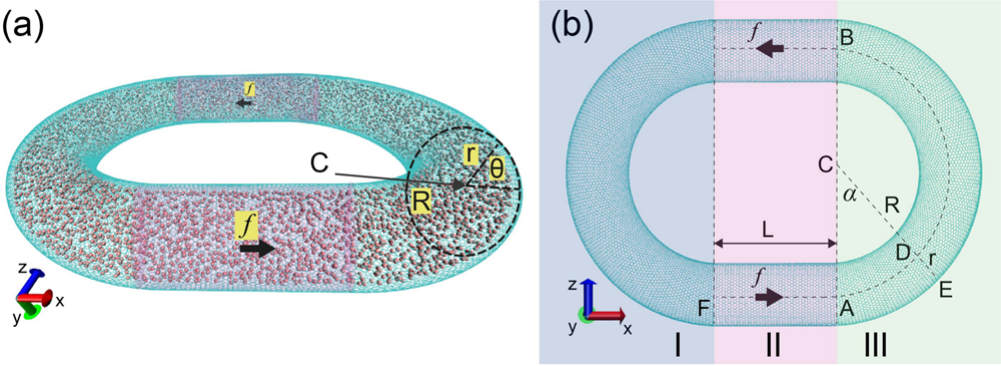
(a) 3D visualization of the curved SWCNT. (b) 2D visualization from the *y* direction. *L* is the length of the straight region for accelerating methane molecules, the bending radius of the channel is *R*, and the tube radius is *r*, α and θ are two angle coordinates for defining the torus surface of the curved CNT.

The circular periodic nanochannel of single-walled carbon nanotube (SWCNT) constructed for this study is depicted in Figure 2. In Figure 2a, a three-dimensional visualization showcases the torus-like nanochannel, colored in cyan, with red balls representing methane molecules. Each methane molecule is represented by a single ball. The torus structure seamlessly connects the head and tail of this CWCNT, ensuring complete confinement of methane molecules. This unique design allows for the periodic flow of methane molecules along the channel, and notably, the angle between the entering velocity and the exiting velocity is π. Consequently, a cubic simulation box with periodic boundary conditions, as employed in most previous studies, is not required. The torus configuration facilitates a continuous and confined flow, eliminating the need for artificial periodicity and enhancing the realism of the simulated nanoscale environment.

In Figure 2a, a distinct straight region is highlighted in pink at the center of the torus-like nanochannel. Methane molecules within this straight segment experience an external force, denoted as *f*. Importantly, the two straight regions flanking the highlighted portion exert forces in opposite directions, imparting a net force that propels the methane within the nanochannel in a counterclockwise direction. Adjacent to this straight region, on both sides, are two halves of a torus structure comprising curved single-walled carbon nanotubes (SWCNTs). These curved sections exhibit a bending radius of *R* and a tube radius of *r*.

In Figure 2b, a comprehensive 2D view of the nanochannel from the *y* direction is presented. The entire channel is systematically divided into three distinct sections. Section II, highlighted for clarity, is a straight segment strategically designed for accelerating methane molecules when the flow velocity is nonzero. Importantly, sections I and III exhibit symmetry, prompting our focused analysis and calculations within section III.

The torus, centrally positioned at *C*, boasts a bending radius of R = *CD*. The geometry of the single-walled carbon nanotube (SWCNT) is characterized by a geometry type vector (*n*, 0),^69^ with a corresponding radius denoted as *r*. Notably, an increase in the value of *n* correlates with a larger radius. Two angles: α, θ that define the torus are shown in Figure 2a,b. When setting the center *C* as the origin (*C*_*x*_, *C*_*y*_, *C*_*z*_) = (0, 0, 0), the surface of section III of this curved SWCNT can be precisely expressed as^79^
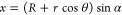
1
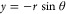
2
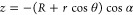
3where α ∈ [0, π], θ ∈ [0, 2π], and *R* > *r*. Nano channel with smaller *R* and *r* would have larger curvatures, and the Gaussian curvature of the 2D surface is give by^80^
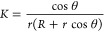
4In the inner half of the surface (π/2 < θ < 3π/2), *K* is negative, while in the outer half (0 < θ < π/2 or 3π/2 < θ < 2π), *K* is positive. And saddle points appear at locations with θ = π.

In this research, the length of the straight region in section 2 is fixed as *L* = 80.0 Å, and we would investigate methane fluid behavior with different *R* and *r*, the tube radius *r* of the CNT depends on *n* in the geometry type vector (*n*, 0), *n* equals 15, 30, 60 and they result in *r* ≈ 5 Å, 10 Å, 20 Å, respectively. The curved nanochannel we focus on is the arc connecting the entrance (point *A*) and the exit (point *B*), the distance between *A* and *B* is . Because *R* > *r*, when *r* = 5 Å, R = (20, 40, 60, 70, 80, 90)Å; when *r* = 10 Å, R = (40, 60, 90)Å; when *r* = 20 Å, R = (60, 90)Å. Parameters for all simulations are list in Table 1.

**Table 1:** Simulation Parameters for Each Run in This Research^a^

ID	*r* (Å)	*R* (Å)	no. of methane	*f* ((kcal/mol)/Å)
1	5.0	20.0	142	-
2				0.01
3		40.0	205	-
4				0.0144
5		60.0	266	-
6				0.0188
7		70.0	300	-
8				0.021
9		80.0	330	-
10				0.0232
11		90.0	360	-
12				0.0254
13	10.0	30	1030	-
14				0.0144
15		60.0	1350	-
16				0.0188
17		90.0	1830	-
18				0.0254
19	20.0	60.0	5000	-
20				0.0188
21		90.0	6800	-
22				0.0254

a “-” in *f* column indicates that the external field in is set to 0.

In this research, we choose to use single-walled carbon nanotubes (SWCNT) to model the nanochannels in kerogen based on the following considerations: First, the cross-sectional shape of SWCNT is circular, which is also the most commonly observed shape in realistic kerogen nanochannels, as determined by experiments. Second, the attractive potential energy between the CNT walls and methane molecules is similar to that between real kerogen and methane molecules, making SWCNT a suitable analog.^76^ Third, SWCNTs are straightforward to construct in simulations, and their structural parameters can be precisely controlled. This control is beneficial for quantitatively investigating the impact of channel geometry on methane behavior. Fourthly, SWCNTs are more accessible for experimental investigations. Realistic nanochannels in kerogen have complex geometries with variable radii and curvatures that are challenging to control. In contrast, the parameters of CNTs can be manipulated in experiments.^69^ Moreover, CNTs can serve as nanocontainers or nanoreactors for various other molecules.^60,70,71^ Therefore, the insights gained from studying CNTs in this paper will also be valuable for other applications and research involving CNTs.

It is crucial to emphasize that, despite employing single-walled carbon nanotubes (SWCNTs) as a modeling framework for nanochannels within porous media, such as the shale matrix, our constructed model remains a simplified representation. In reality, nanochannels in shale matrices typically exhibit rough surfaces and contain numerous disordered aromatic molecules and functional groups. These complexities introduce additional intricacies to the fluid–solid interactions within the nanochannels.

It is pertinent to note that our research primarily focuses on elucidating the effects of curved structures on methane nanofluid dynamics. As such, we deliberately opt for a simplified SWCNT model to isolate and investigate the impacts of the curved geometry. While acknowledging the simplifications made in our model, these choices enable a more targeted exploration of the specific influence of nanochannel curvature on methane flow, allowing us to draw meaningful insights without the confounding effects of additional factors present in realistic shale matrix nanochannels. After gaining sufficient knowledge about the impact of curvature on methane transportation, we can gradually add more complexities to model the nanochannel. These complexities could include adding functional groups or roughness to the surface of the walls, altering the shape of the cross-section, or even constructing the curved nanochannel by compressing multiple individual kerogen molecules.^63,77,81^ This progressive approach will allow us to build a more comprehensive and accurate model of the nanochannels in kerogen, enhancing our understanding of methane behavior in these environments.

### 2.2 Molecular Dynamics Simulation

The molecular dynamics simulations were executed utilizing the Large-scale Atomic/Molecular Massively Parallel Simulator (LAMMPS)^82,83^ operating under the canonical ensemble (NVT). To emulate conditions akin to those found deep underground in shale formations, the system temperature was set to 400 K. Temperature control was maintained through coupling with a Nosé–Hoover thermostat featuring a damping parameter of 0.1 ps. Furthermore, the number of methane molecules in each simulation was meticulously optimized to ensure the system pressure approximated 30 MPa, replicating realistic shale conditions, and similar temperatures and pressures are widely used to study shale gas properties in a number of other researches.^48,63,77^ Note that, given the canonical ensemble (NVT) conditions, there is no explicit pressure coupling, resulting in the actual pressure fluctuating around the targeted value. The Velocity-Verlet algorithm, with an integration step time of 2 fs, was employed to generate simulation trajectories.

All simulations were performed on 4 × 5.2 GHz Intel i9–12900KF cores and accelerated by 1 × NVIDIA GeForce RTX 3080 Ti GPU card. The structure of the curved SWCNT is constructed using in-house python code. The methane molecule is modeled as a single united atom. The final topology was assembled and constructed using Gromacs,^84^ and then transferred to a LAMMPS structure file using the Gro2lam package.^85^

There are two types of atoms in all simulations: C_*Aro*_ is the aromatic carbon atom in the SWCNT, CH_4_ is the single united atom that represents a methane molecule. The interaction between atom type *i* and *j* is determined using the Lennard-Jones function:
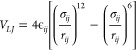
5with a cutoff of 1.2 nm, where *r*_*ij*_ is the distance between atom *i* and *j*. Values of ϵ_*ij*_ and σ_*ij*_ are list in Table 2. When an atom is interacting with another one with the same type, we have *i* = *j*, and ϵ_*ii*_ = ϵ_*i*_, σ_*ii*_ = σ_*i*_. If *i* ≠ *j*, we use geometrical rules to obtain parameters for the interaction between two different atom types:
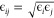
6
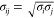
7

**Table 2:** Parameters of the Lennard-Jones Functions for Each Atom Type Used in This Research

atom	ϵ (kcal/mol)	σ (Å)	mass (Da)
C_*Aro*_	0.07342	3.52053	12.0110
CH_4_	0.30187	3.70995	16.0430

The ϵ_*ij*_ and σ_*ij*_ parameters utilized in our simulations are derived from the Gromos54a7 force field,^86^ a well-established force field particularly suited for simulating hydrocarbons and biomolecules. Given that the SWCNT and methane united atoms are electro-neutral, and the SWCNT is held in a frozen state while only allowing the movement of methane molecules, electrostatic interactions in our system can be safely ignored.

To ascertain the accuracy of the interaction parameters employed, we conducted additional simulations involving methane gas in the bulk state at *T* = 400 K, under a pressure of 30 MPa. The resulting density from our simulations was found to be 8.688 mol/L. In comparison, experimental measurements yielded a density of 8.793 mol/L.^87^ The relative error between our simulation and experimental data is a mere 1.19%. This small error serves as a robust validation, affirming that the force field parameters in our model accurately capture the properties of methane under the high-pressure and high-temperature conditions representative of subsurface shale environments.

Two types of MD simulations are carried out, the first type is equilibrium MD, where the flow velocity is zero, which is used for studying the adsorption of methane fluid onto the inner wall of SWCNT; the second type is nonequilibrium MD,^48,52,53,67^ especially, we are using the external field nonequilibrium MD (EF-NEMD), which has been widely used in the simulation of nanofluid in nanochannels, mostly been applied in straight channels.^48,52,53,57,67^ In straight channels, the whole system is been accelerated, the pressure difference between two ends of the channel in the periodical simulation box can be rigorously obtained tobe^67,77^
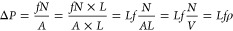
8where *f* is the force added to each molecule, and *N* is the totally number of molecules in the system, *A* is the area of the cross section, *L* is the length of the channel, and the pressure gradient could be obtained as
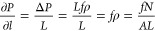
9

If molecules in only part of the channel are exerted with forces, pressure difference in the channel is still the same as in eq 8, but *N* should be the number of molecules with external force in the accelerated part, rather than all molecules, *V* now becomes the volume of part of the channel with external forces. The length with external force is *L*, and the whole length of the channel in periodic box is *L*_0_, the pressure gradient could be obtained as
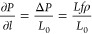
10Note that in this case, *L* < *L*_0_, since *L* is only part of the whole channel.

In our system, the acceleration region is part of the whole channel, so we should refer to eq 10, and the whole channel contains two periodically identical sections, each section has one acceleration part of length *L*, plus one curved half (section I or III) whose length is *πR*, which means *L*_0_ = *L* + *πR*, and eq 10 is true for both replication, because they are periodically symmetrical to each other, so the pressure gradient in the entire channel is
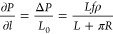
11this pressure gradient ∂*P*/∂*l* could push methane fluid flow in the SWCNT in the counterclockwise direction. From eq 11 we can see that both *f* and *R* could impact ∂*P*/∂*l*, and in this research, we will set *f*_0_ = 0.01(kcal/mol)Å when *R* is the minimal, and R = *R*_0_ = 20 Å. When *R* is larger, we would ensure that the ∂*P*/∂*l* to be the same for all situations, so that the force *f* for any other *R* can be determined by the eq 13 as follows, and all corresponding forces *f* are listed in Table 1.
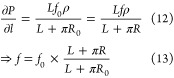
12

Each simulation spans a duration of 6000 ps. To ensure the stability of the system and maintain a constant flow velocity, the initial 1000 ps is dedicated to equilibration. Subsequently, the remaining 5000 ps trajectory is utilized for in-depth analyses.

## 3 Results and Discussions

### 3.1 Methane Adsorption in the Curved SWCNT

In the initial phase of our investigation, equilibrium simulations are conducted, where no external force is applied to methane molecules. Utilizing the equilibrium molecular dynamics (MD) trajectories, we compute the average methane adsorption number density distribution (ρ) within the 2D cross section of the SWCNT. This 2D space is defined by the coordinates ξ and *y*, where ξ signifies the radial coordinate perpendicular to *y* within the cross section.

**Figure 3:**
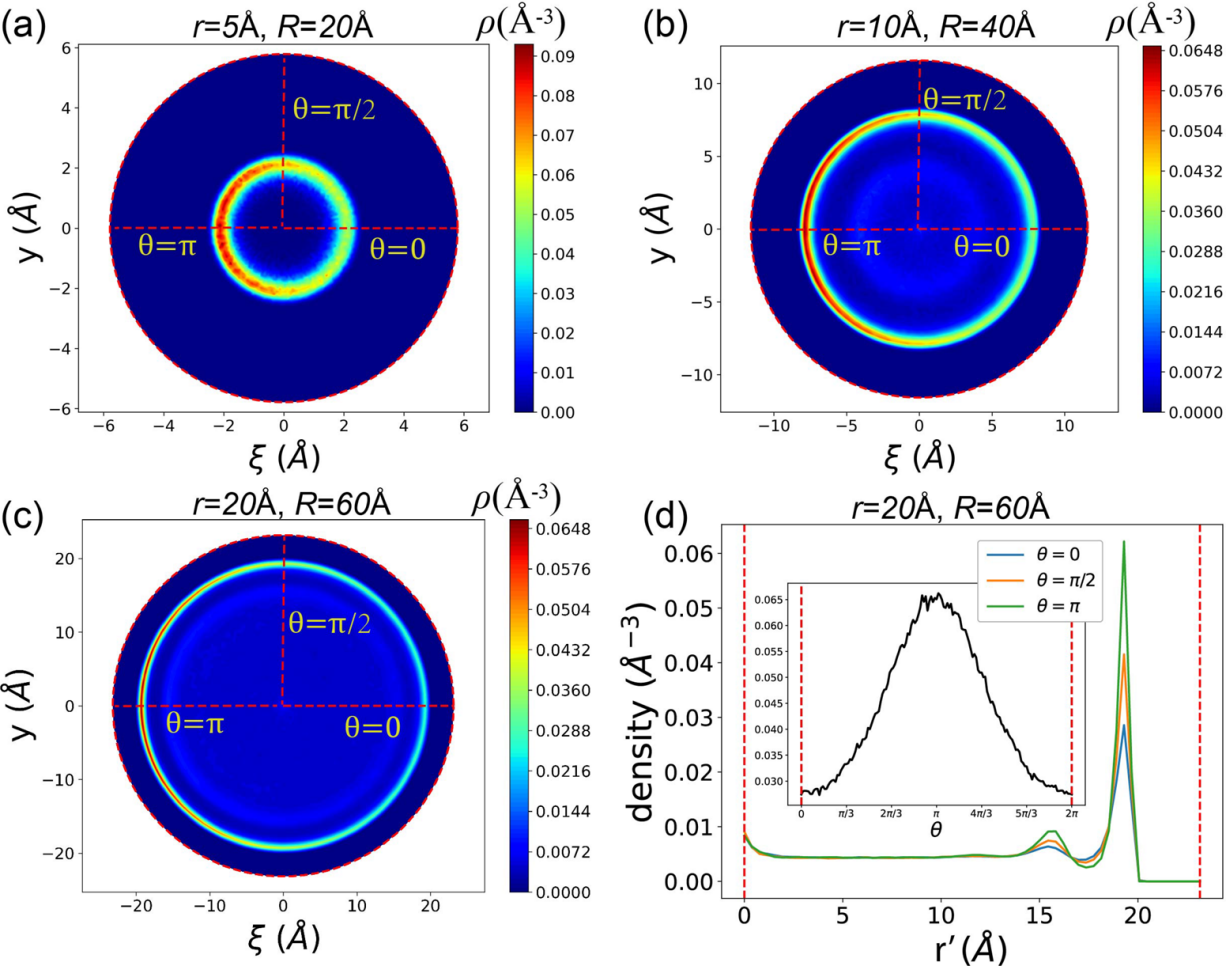
Adsorption densities shown in 2D cross section (ξ, *y*) space for the case of (a) (*r*, *R*) = (5 Å, 20 Å), (b) (*r*, *R*) = (10 Å, 40 Å), (c) (*r*, *R*) = (20 Å, 60 Å). Red dashed lines indicate θ = 0, π/2, π. Red dashed circle indicates the surface of the SWCNT. (d) The adsorption density as a function of radial coordinate *r*′ for three typical θs in the case of (*r*, *R*) = (20 Å, 60 Å). The inset shows the relation between the adsorption peak and θ in the same case.

Figure 3a–c illustrates the distributions of ρ(ξ, *y*) for varying tube radii (*r* = 5 Å, 10 Å, 20 Å). Notably, methane molecules are observed to distribute exclusively within a circular layer, with the maximum adsorption density concentrated near the inner region (θ = π). This observation suggests that the negative curvature of the SWCNT exhibits a stronger attraction to methane molecules. It is worthy to be clarified that the density ρ(ξ, *y*) is calculated as
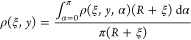
14which is the averaged number density displayed in the (ξ, *y*) space, obtained by taking the average of the 3D number density ρ(ξ, *y*, α) over the arc along the nanochannel with α ranging from 0 to π, so its unit is still Å^–3^.

Additionally, a distinct gap emerges between methane molecules and the SWCNT wall surface, characterized by a density of 0.0. The width of this gap measures approximately 3.0 Å. This gap can be attributed to the repulsive interaction between methane molecules and the SWCNT, particularly at small distances. The presence of this gap underscores the delicate balance between attractive and repulsive forces, influencing the spatial distribution of methane within the SWCNT during equilibrium conditions. This phenomenon is also observed by previous studies.^76^

To examine the evolution of adsorption density along the radial coordinates *r*′ and θ within the tube, we focus on the case where (*r*, *R*) = (20 Å, 60 Å). Figure 3d illustrates the relationship between ρ and *r*′ along three distinct radial directions: θ = 0, π/2, π. Notably, all three density distributions exhibit a prominent adsorption peak near the surface (*r*′ ≈ 20 Å). Note that the radius for the wall is *r*′ ≈ 23 Å, the width of the zero density gap is about 3.0 Å.

When θ = π, the adsorption peak is maximized, and an additional lower secondary peak becomes apparent at *r*′ ≈ 15 Å. The inset of Figure 3d displays the relationship between peak heights and θ using the black curve. This curve illustrates a continuous increase in peak height from 0.03 Å^–3^ to 0.065 Å^–3^ as θ ranges from 0 to π. This observation suggests a correlation between the angular position within the SWCNT cross-section and the adsorption density, highlighting the influence of curvature on the spatial distribution of methane molecules within the nanotube.

**Figure 4:**
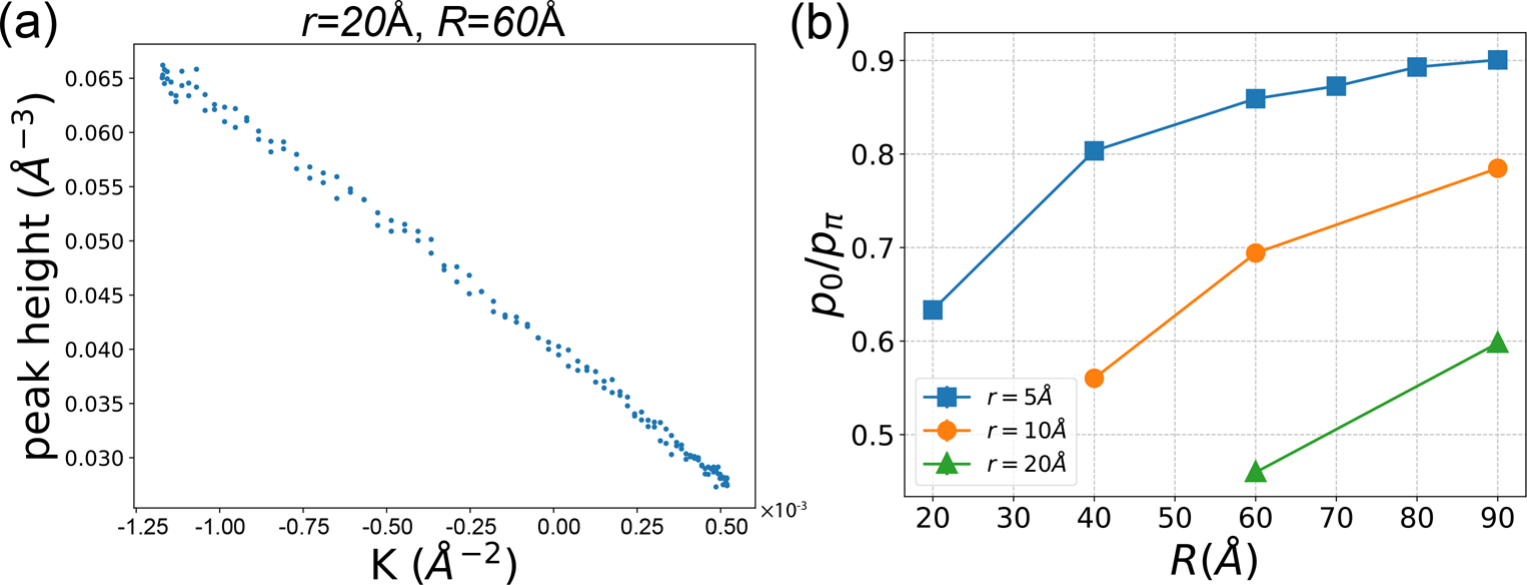
(a) The relation between the adsorption peak height and Gaussian curvature in the case of (*r*, *R*) = (20 Å, 60 Å). (b) The ratio of the adsorption peak *p*_0_/*p*_π_ for all *r*s and *R*s.

The adsorption peak height *p*(θ) exhibits a strong negative correlation with the Gaussian curvature *K*(θ), as defined in eq 4. This relationship is illustrated in Figure 4a. The negative correlation suggests that the ability of a surface to attract methane is significantly influenced by its curvature. Specifically, smaller curvature corresponds to stronger adsorption within the SWCNT, and this behavior is also reported by previous studies.^88−90^

One prominent observation is the variation in adsorption peak heights along the radius, particularly at θ = 0 and θ = π. To quantify this discrepancy, we introduce the ratio of the two peak heights, denoted as *p*_0_/*p*_π_. Here, *p*_0_ and *p*_π_ represent the peak heights along θ = 0 and π respectively.

Figure 4b illustrates *p*_0_/*p*_π_ for different combinations of *r* and *R*. Notably, all *p*_0_/*p*_π_ ratios are smaller than 1.0. As *R* increases, *p*_0_/*p*_π_ exhibits a rising trend, approaching 1.0. This trend is attributed to the fact that, when *r* is fixed, increasing *R* makes the nanochannel more akin to a straight carbon nanotube (CNT) with infinite *R*. Consequently, the discrepancy between the inner and outer surfaces diminishes, leading to *p*_0_/*p*_π_ approaching 1.0 for larger *R* values.

Conversely, as *r* increases, *p*_0_/*p*_π_ decreases toward 0. This behavior is explained by the fact that, with a fixed *R*, increasing *r* results in a flatter outer surface and a more twisted inner half. This discrepancy in surface characteristics intensifies, leading to a smaller *p*_0_/*p*_π_ ratio.

### 3.2 Velocity and Flux of Methane Flow

When each methane molecule experiences an external force *f*, a positive pressure gradient is induced within the nanochannel, specifically in the counterclockwise direction. This pressure gradient serves as the driving force for gas flow along the channel. To maintain consistency across simulations, *f* is adjusted based on the channel length, ensuring that the pressure gradient ∂*P*/∂*l* remains the same for all cases with different *R*.

**Figure 5:**
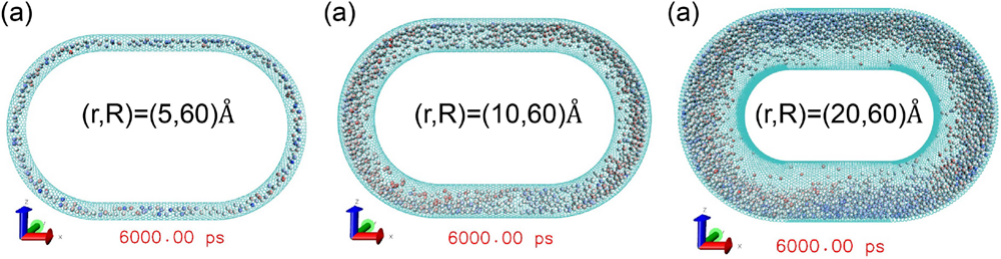
Snapshots of the final step in EF-NEMD simulation of the methane flow in the curved SWCNT, for the case of (a):(*r*, *R*) = (5, 60)Å, (b):(*r*, *R*) = (10, 60)Å, and (c):(*r*, *R*) = (20, 60)Å. Methane molecules are colored with their velocities, red indicate small velocities while blue indicate large velocities.

Figure 5 provides snapshots of the final step in three representative simulations. These simulations correspond to cases where *r* = 5, 10, 20 Å and R = 60 Å and corresponding videos can be found in the Supporting Information. These visual representations offer insights into the dynamic behavior of methane gas flow within the nanochannel under the influence of the external force.

**Figure 6:**
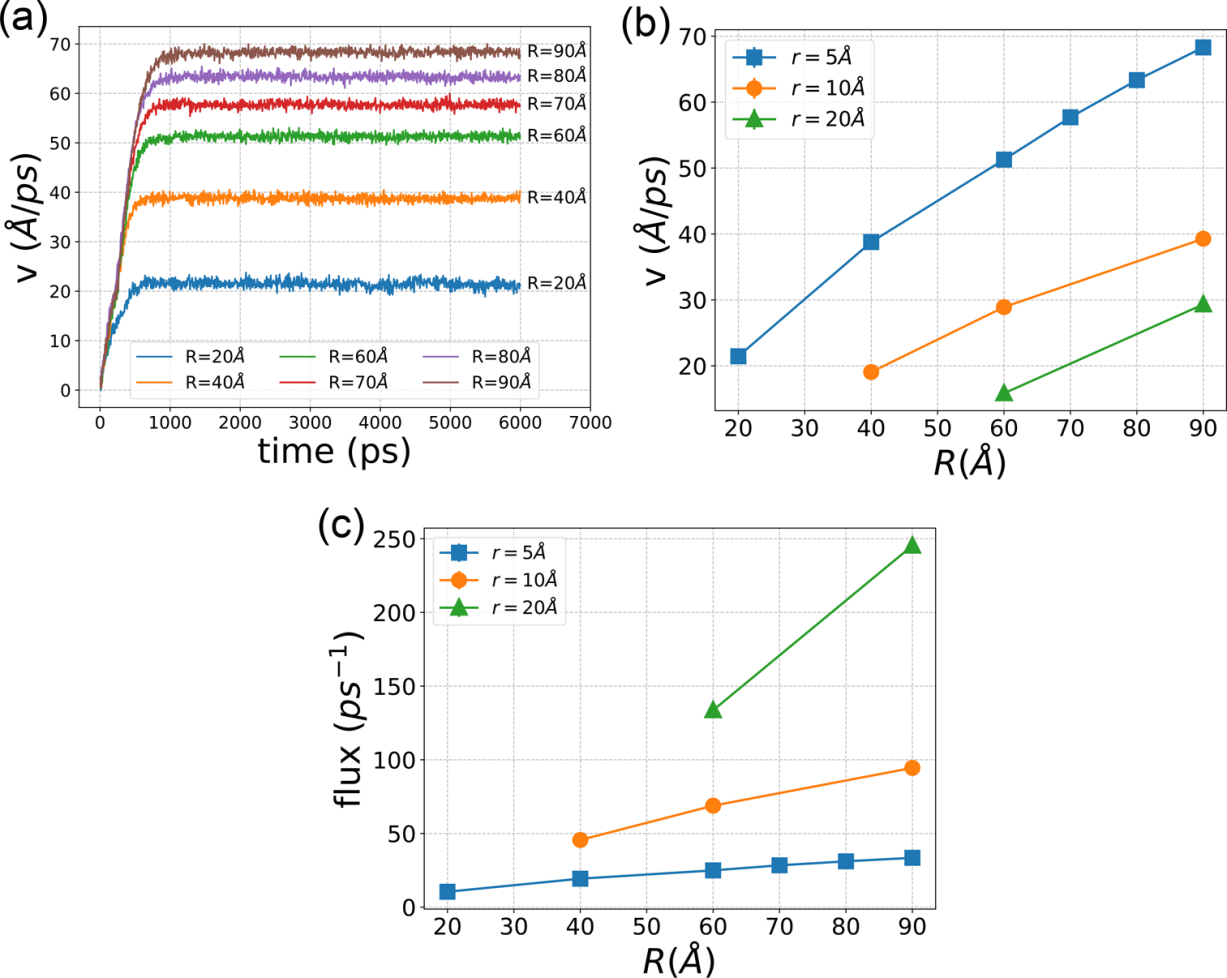
(a) Time evolution of the average fluid velocity in the straight region for the case of *r* = 5 Å and different *R*s. (b) Stabilized fluid velocities for all (*r*, *R*). (c) Fluid flow flux for all (*r*, *R*).

Figure 6a presents the time evolution of methane velocity along the channel within the straight region II for channels with *r* = 5 Å and different *R* values. The observation reveals that, over time, velocities gradually increase from 0 to reach distinct plateaus, indicating stable and unchanging final velocities. The time required for fluid velocity to stabilize is consistently less than 1000 ps across different *R* values. Notably, as *R* increases, the stabilization time experiences a slight increase, while the stabilized fluid velocity attains higher values. This phenomenon is also observed by previous studies.^68^ The flow velocity in CNT is more than 10 times larger than the velocity in rough nanochannels,^77^ due to the smoothness of the inner walls of CNT, as shown in previous theoretical and experimental researches,^60,70,71,75^ and our previous studies also show that methane flow in nanochannel constructed by smooth graphene has much higher velocities than that in rough nanochannel.^66^

This behavior is attributed to the fact that, with an increase in *R*, the curved nanochannel more closely resembles a straight carbon nanotube (CNT). Consequently, the impediment from the bended wall, especially when the flow changes direction, is reduced. This reduction in impediment contributes to a faster stabilization of fluid velocity and an overall increase in the stabilized velocity for larger *R* values.

Figure 6b presents the stabilized velocities for various combinations of *r* and *R*. A consistent observation is that, for all *r* values, the stabilized velocity experiences an increase with an increase in *R*. Additionally, the trend reveals that larger tube radii (*r*) correspond to smaller stabilized velocities.

This trend can be attributed to the emergence of strong turbulent flows, especially vertical to the tangent velocity, in channels where *r* is large. In contrast, narrower channels exhibit weaker turbulence. The presence of strong turbulence in wider channels acts as a counterforce, reducing fluid velocities. Consequently, the stabilized velocity is smaller when *r* is larger. There will be deeper discusses of this finding in section 3.4.

Figure 6c illustrates the flux of the fluid, defined as the number of methane molecules passing through the cross section per unit time, for various combinations of (*r*, *R*). A clear observation is that, for a fixed *r*, the flux increases with an increase in *R*. This trend is attributed to the higher transport velocities observed in channels with larger bending radii (*R*).

Conversely, for a fixed *R*, the flux increases with an increase in *r*. Despite the lower velocities of methane fluid in wider channels, the larger cross-sectional area compensates for the effect of lower velocity. As a result, wider channels with larger *r* facilitate more methane flow across the cross section per unit time.

### 3.3 Impacts of the Flow toward Adsorption

**Figure 7:**
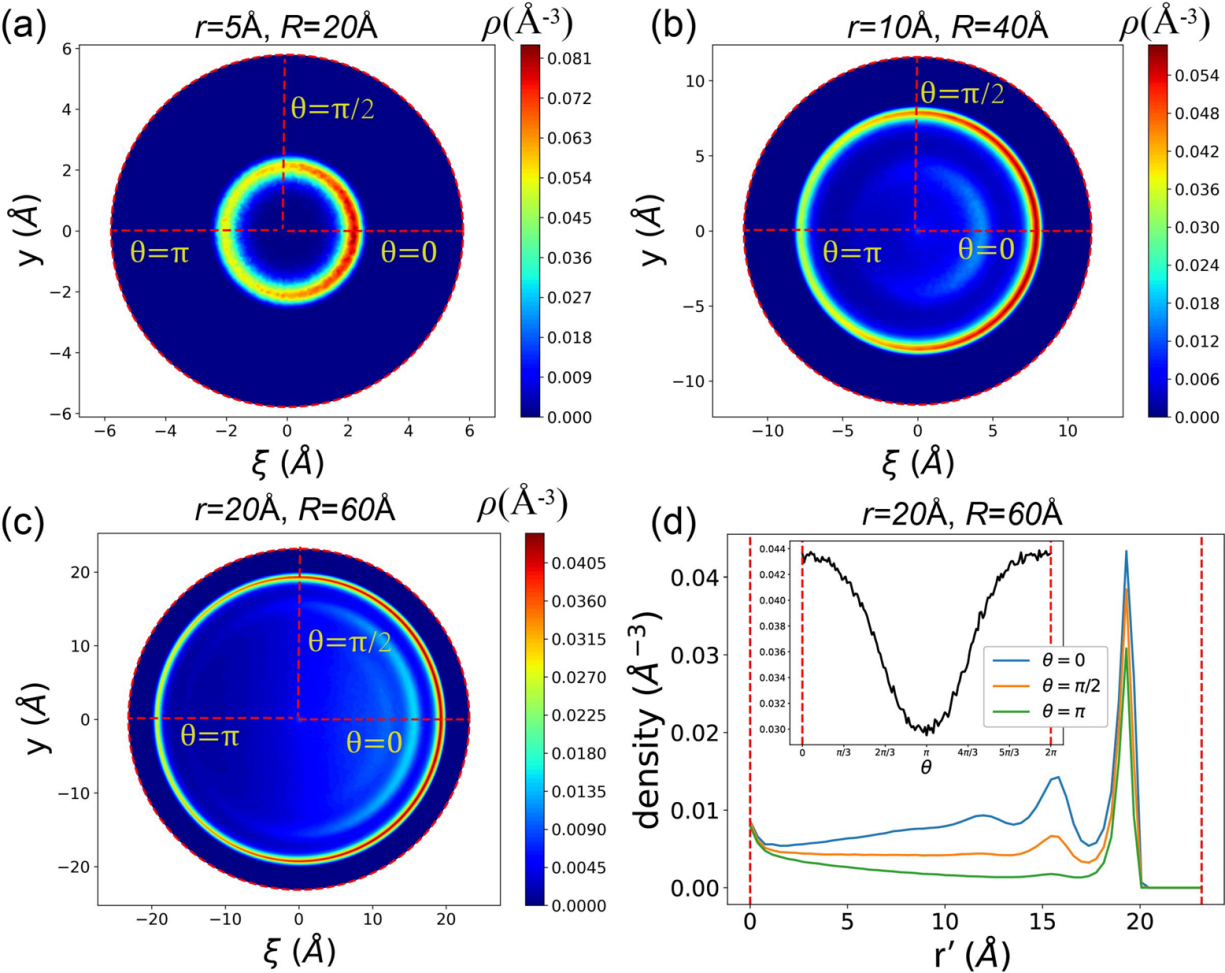
When the methane flow is turned on, adsorption density shown in 2D (ξ, *y*) space for the case of (a) (*r*, *R*) = (5 Å, 20 Å), (b) (*r*, *R*) = (10 Å, 40 Å), (c) (*r*, *R*) = (20 Å, 60 Å). Red dashed lines indicate θ = 0, π/2, π. Red dashed circle indicates the surface of the CNT. (d) The adsorption density as a function of the radial coordinate *r*′ for three typical θs in the case of (*r*, *R*) = (20 Å, 60 Å). The inset shows the relation between the adsorption peak and θ in the same case.

Figure 7a–c illustrates the adsorption density in the (ξ, *y*) space for three different combinations of tube and bending radii (*r*, *R*). Notably, the location of the maximum adsorption density shifts from θ = π to θ = 0 when the flow is turned on. This shift is attributed to the inertial motion of methane fluid toward the tangent direction. In the presence of a bending SWCNT, the flow tends to be squeezed in the outer region, resulting in higher density at θ = 0 compared to θ = π.

For the specific case of (*r*, *R*) = (20 Å, 60 Å), Figure 7d showcases the adsorption density as a function of *r*′ along the radius for three different θ values (θ = 0, π/2, π). The inset of Figure 7d presents the peak height as a function of θ. A notable observation is that, compared to Figure 3d where the flow is turned off, the order of peak height is reversed when the flow is turned on. The largest adsorption density now occurs at θ = 0, emphasizing the significant impact of fluid flow on the spatial distribution of methane adsorption within the nanochannel.

**Figure 8:**
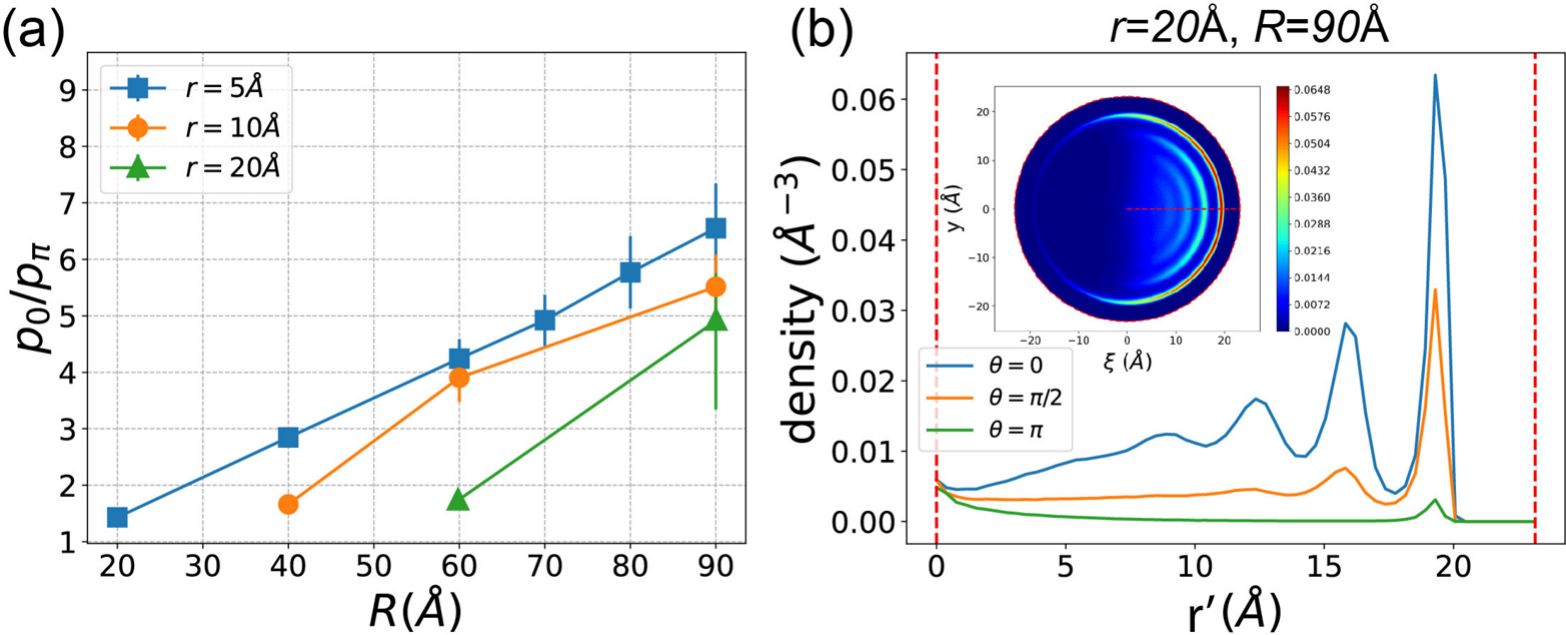
(a) When the flow is turned on, the ratio of the adsorption peak *p*_0_/*p*_π_ for all *r*s and *R*s. (b) The adsorption density as a function of the radial coordinate *r*′ for three typical θs in the case of (*r*, *R*) = (20 Å, 90 Å). The inset shows the 2D adsorption density in the (ξ, *y*) space.

Figure 8a illustrates the ratio (*p*_0_/*p*_π_) for various combinations of tube and bending radii (*r*, *R*). All *p*_0_/*p*_π_ values are greater than 1.0, indicating that, due to the nanoflow, adsorption at the outer surface is favored over the inner surface. As *R* increases, *p*_0_/*p*_π_ also increases. This is because the flow velocity is higher in nanochannels with larger *R*, as mentioned in section 3.2, thereby enhancing the trend of methane fluid being squeezed at the outer surface. As shown in Figure 8b, where R = 90 Å, compared to Figure 7c and d, where R = 60 Å, methane fluid is significantly tend to be locate at the outer surface. The same reasoning applies to the case of smaller *r*: the ratio increases with a decrease in *r*, because the flow velocity in narrower channels is also larger.

### 3.4 Flow Velocity Distribution in the Curved SWCNT

**Figure 9:**
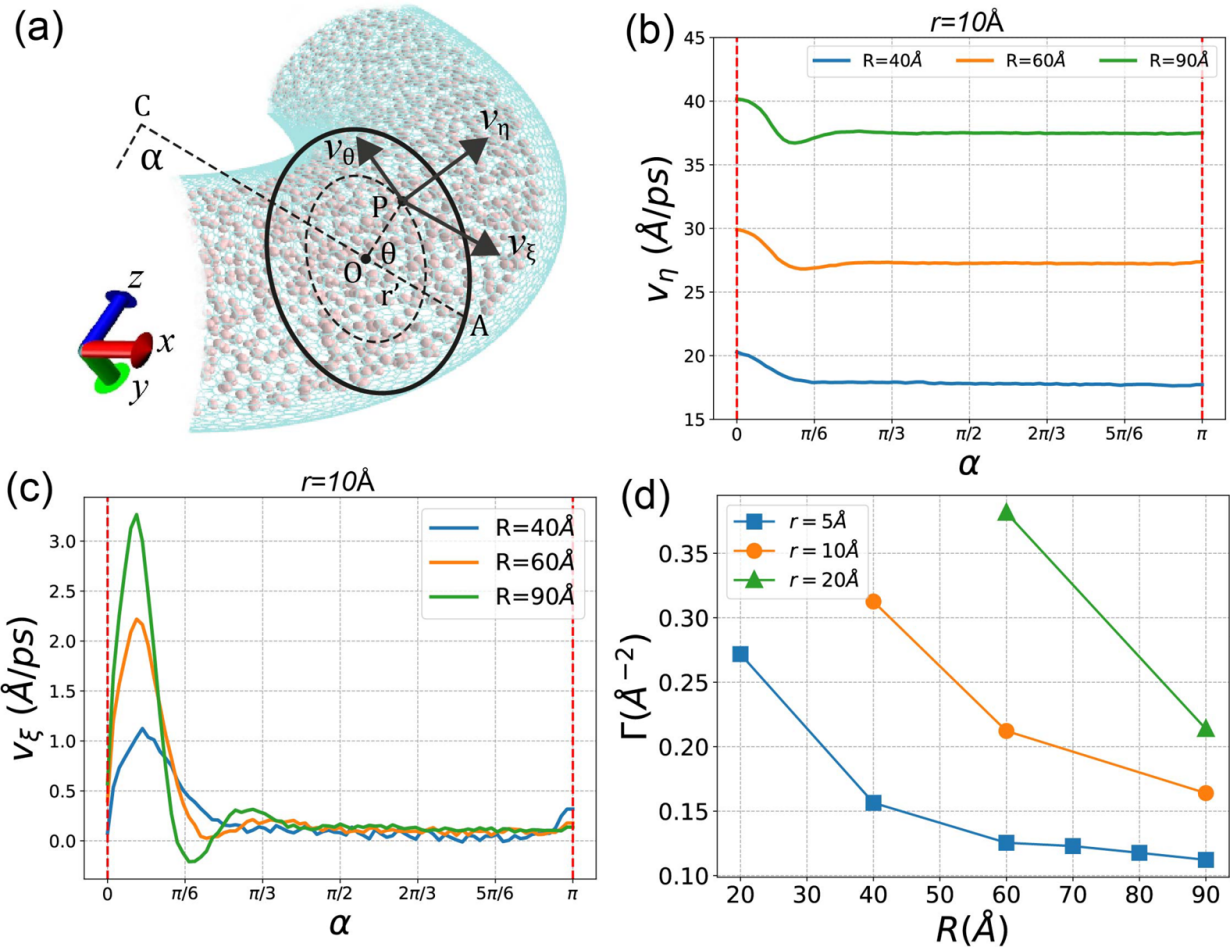
(a) *v*_η_, *v*_ξ_, *v*_θ_ for the point P in the nanochannel. (b) Tangent transport velocity *v*_η_ as a function of α for the case of *r* = 10 Å and different *R*s. (c) The vertical velocity *v*_ξ_ as a function of α for the cse of *r* = 10 Å and different *R*. (d) The degree of turbulence Γ for all *r*s and *R*s.

In this section, we will discuss the distribution of different velocity components in the nanochannel, these components include the tangent velocity *v*_η_ along the nanochannel, the vertical velocity *v*_ξ_, and the radial velocity *v*_θ_, which is along the tangent direction in the (*r*′, θ) space, *v*_η_, *v*_ξ_, *v*_θ_ are shown in Figure 9a and are defined as in the following equations:
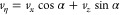
15
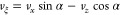
16
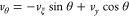
17

For each methane molecule within a frame, we can calculate its *v*_η_, *v*_ξ_, *v*_θ_ components based on *v*_*x*_, *v*_*y*_, *v*_*z*_. Subsequently, the average velocity at a specific location *P*(α, *r*′, θ) inside the channel is determined as the mean of all methane velocities at that location throughout the entire simulation.

In Figure 9b, the tangent velocity *v*_η_ is presented as a function of α for three different *R*s when *r* = 10 Å. As previously discussed, larger *R*s correspond to higher flow velocities. Notably, at the entrance of the curved nanochannel, where α = 0, there is a discernible velocity drop. This drop is attributed to the sudden change in the curvature of the nanochannel, inducing turbulence in the entrance region. This turbulent effect is further illustrated in the plot of vertical velocities *v*_ξ_ in Figure 9c.

In Figure 9c, a notable velocity pulse is evident near the entrance where 0 < α < π/6. Beyond α > π/6, *v*_ξ_ rapidly diminishes after a few minor oscillations, with larger *R* exhibiting a higher pulse peak. The magnitude of turbulence can be quantified by this pulse peak height, where a larger turbulent effect corresponds to a higher pulse peak. However, it is essential to consider that a larger fluid tangent velocity *v*_η_ could also result in a higher *v*_ξ_ pulse peak. To obtain a more representative measure, we define the ‘relative degree of turbulence’ as the ratio of the pulse peak value to the stabilized tangent velocity *v*_η_ (as shown in Figure 6b), denoted as Γ.

In Figure 9d, Γ values for all *r*s and *R*s are presented. It is evident that smaller *r* values correspond to a lower degree of turbulence. This suggests that the methane fluid can transition from the straight region (II) into the curved channel more smoothly, experiencing less hindrance from the abrupt change in curvature. This observation aligns with the earlier findings in Section 3.2, where narrower nanochannels exhibited faster fluid velocities.

Additionally, in Figure 9d, it is noteworthy that smaller *R* values result in a larger degree of turbulence. This can be easily understood: smaller *R* values lead to larger curvatures in the bended SWCNT, as indicated in eq 4. The significant change in curvature during the transition from the straight section to the bended section results in a larger degree of turbulence.

**Figure 10:**
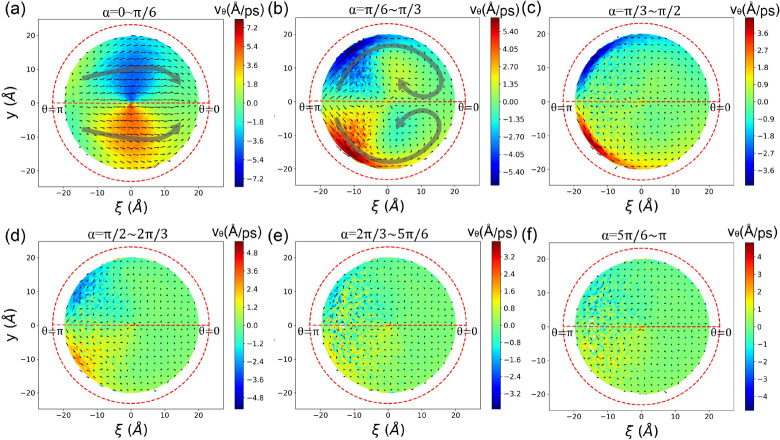
Radial velocity *v*_θ_ in the (ξ, *y*) space from different α sections along the nanochannel. (a) 0 < α < π/6, (b) π/6 < α < π/3, (c) π/3 < α < π/2, (d) π/2 < α < 2π/3, (e) 2π/3 < α < 5π/6, (f) 5π/6 < α < π. Red dashed circle indicate the boundary surface of the CNT. White space inside the boundary means that there is no methane, and we do not calculate *v*_θ_ for these region. Small black arrows in each panel indicate the magnitude and direction of the flow velocity.

In Figure 10, the distribution of the radial velocity *v*_θ_ in the (ξ, *y*) space is visualized, with the *v*_θ_ component depicted in Figure 9a and defined in eq 17. Positive values indicate a counterclockwise flow, while negative values denote a clockwise flow. Figure 10a corresponds to the segment near the entrance where 0 < α < π/6, illustrating fluid flow from the inner half (π < θ < 3π/2) toward the outer half (0 < θ < π/2 or 3π/2 < θ < 2π).

Figure 10b corresponds to the segment with π/6 < α < π/3, where the fluid flows near the top and bottom surfaces of the nanochannel and turns back upon meeting, forming vortex flow. Figure 10c, d, e, f correspond to segments where α > π/3, and turbulence has diminished. The volute pattern in the *v*_θ_ profile weakens as α approaches π.

**Figure 11:**
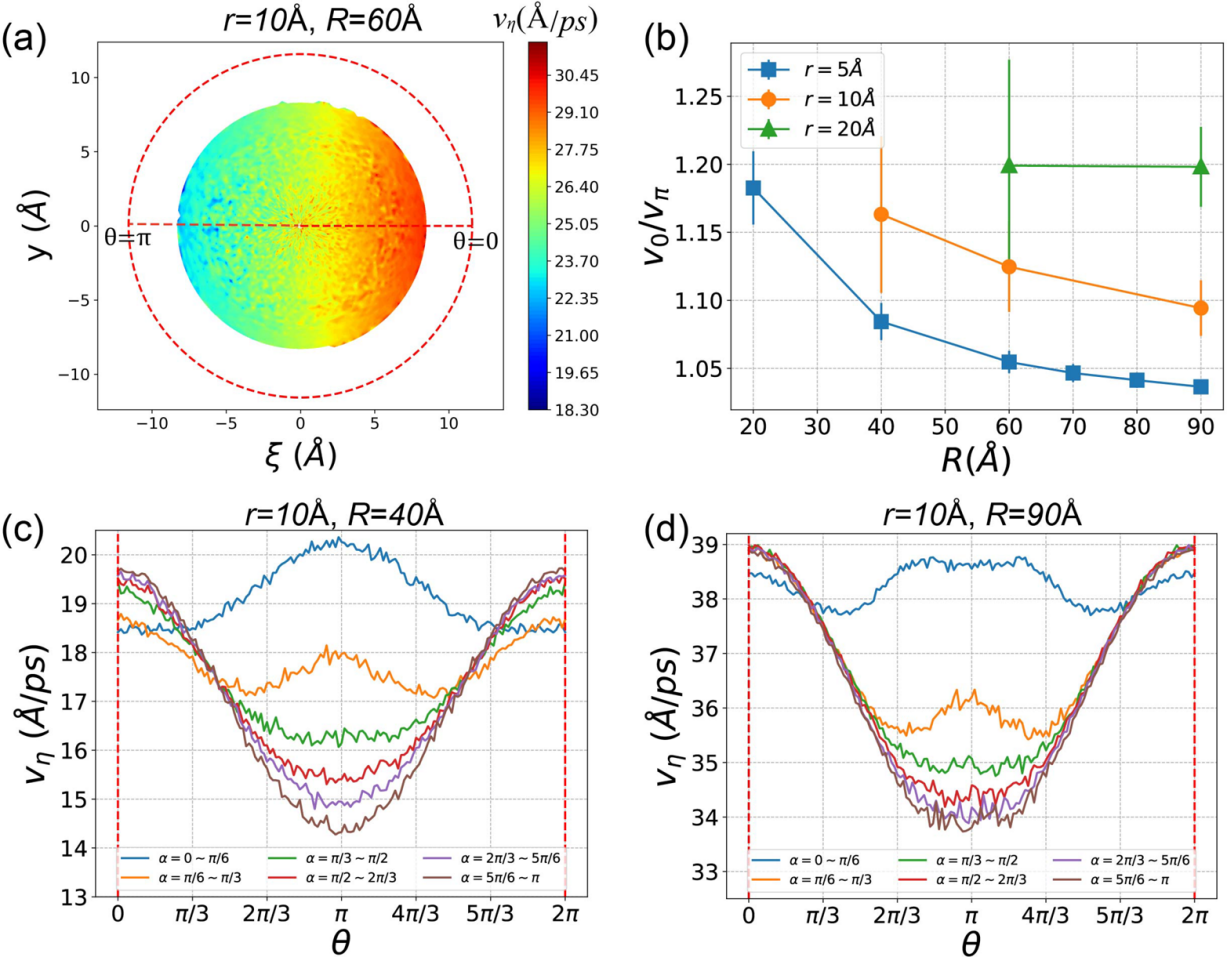
(a) Tangent velocity distribution in the cross section in the (ξ, *y*) space for the case of (*r*, *R*) = (10 Å, 60 Å). Red dashed circle indicate the boundary surface of the CNT. White space inside the boundary means that there is no methane, and we do not calculate *v*_η_ for this region. (b) The ratio between the outer velocity and inner velocity *v*_0_/*v*_π_ for all *r*s and *R*s. Tangent velocity *v*_η_ as a function of θ for different parts along the nanochannel with differernt αs, for the case of (c): (*r*, *R*) = (10 Å, 40 Å) and (d): (*r*, *R*) = (10 Å, 90 Å).

The tangent flow velocity *v*_η_ exhibits a heterogeneous distribution in the (ξ, *y*) space within the nanochannel. Figure 11a illustrates the averaged *v*_η_(ξ, *y*) for the case of (*r*, *R*) = (10 Å, 60 Å). Notably, the tangent transport velocity in the outer region (0 < θ < π/2 or 3π/2 < θ < 2π) surpasses that in the inner region (π < θ < 3π/2). The highest tangent velocity occurs at θ = 0 (denoted as *v*_0_), while the minimum velocity occurs at θ = π (denoted as *v*_π_). The ratio between *v*_0_ and *v*_π_ serves as a characterization of the discrepancy in tangent transport velocity between the outer and inner surfaces.

In Figure 11b, *v*_0_/*v*_π_ for all *r*s and *R*s is presented. Each value represents the average of *v*_0_/*v*_π_ over the curved nanochannel, and the error bars denote the standard errors of *v*_0_/*v*_π_ values from six segments along the nanochannel. Each segment spans an α range of π/6. Notably, as *R* increases, *v*_0_/*v*_π_ decreases. This is attributed to the fact that channels with larger *R* more closely resemble a straight tube, resulting in a smaller velocity discrepancy as in a straight tube. Conversely, *v*_0_/*v*_π_ increases with the widening of the channel (*r*), indicating that wider channels have a more pronounced impact on tangent velocity heterogeneity.

It is worth noting that the error bars for smaller *R*s are larger. To elucidate this, we plotted the averaged tangent velocity as a function of θ, *v*_η_(θ), for six segments along the curved nanochannel with different α. Figure 11c and d represent the cases of (*r*, *R*) = (10 Å, 40 Å) and (10 Å, 90 Å), respectively.

From Figure 11c and d, we observe that *v*_η_(θ) for the first segment (0 < α < π/6) exhibits a bell-shaped distribution, with the maximum velocity at θ = π. As α increases and the flow approaches the exit where α = π, *v*_η_(θ) gradually converges, with the maximum velocity occurring at θ = 0. This implies that the tangent velocity *v*_η_ is not only heterogeneous in the (ξ, *y*) space but also along the curved nanochannel with different α. The distribution at the entrance (α = 0) differs significantly from the converged distribution.

Comparing Figure 11c with 11d, we observe that as α increases, the convergence speed for small *R* is slower than for larger *R*. The *v*_η_(θ) distributions in the six segments are more distinct from each other in the case of smaller *R*, which explains why error bars in Figure 11b are larger for smaller *R*.

## 4 Conclusions

In conclusion, our exploration of methane fluid behavior within curved single-walled carbon nanotubes (SWCNTs) employing external field nonequilibrium molecular dynamics simulation (EF-NEMD) within a circular periodic system has yielded significant findings. The following key conclusions can be drawn from our study:

When the external force is turned off, the adsorption of methane fluid is most pronounced near the inner half surface of the curved SWCNT, where negative Gaussian curvature prevails. Notably, a larger Gaussian curvature results in diminished adsorption strength.

When the external force is turned on, methane will flow in the nanochannel, and nanochannels characterized by a larger bending radius (*R*) exhibit behavior closely resembling that of straight tubes, facilitating higher transport velocities for methane flow. Conversely, an increased tube radius (*r*) induces a decrease in transport velocity due to heightened impediment caused by turbulence at the entrance of the curved nanochannel, where sudden changes in curvature occur. Remarkably, despite the slower transport velocity in nanochannels with larger *r*, the flow flux remains larger, underscoring the complex interplay between curvature and radius effects on methane transport dynamics.

Upon initiation of the flow, a notable shift in methane adsorption properties is observed. Specifically, the outer half surface manifests a heightened adsorption capacity compared to the inner half surface. This phenomenon arises from the fluid’s inertial motion, prompting a natural tendency to move away from the curved channel. Consequently, an increased adsorption density is observed near the outer surface, underlining the dynamic interplay between fluid motion, channel curvature, and adsorption characteristics.

We also investigate the flow velocity distribution in the nanochannel: At the entrance of the nanochannel, the tangent flow velocity experiences deceleration due to the abrupt change in curvature. A distinct vertical velocity pulse can be observed, which initiates turbulent vortex flow. Notably, this phenomenon is more pronounced in channels characterized by smaller bending radius (*R*) and larger tube radius (*r*). Furthermore, the distribution of tangent flow velocity within the nanochannel is heterogeneous. Specifically, velocities are higher near the outer half surface and slower near the inner half surface. However, it is noteworthy that this relationship is inverted in the entrance region. These observations underscore the intricate flow dynamics influenced by nanochannel geometry and curvature variations.

Also need to be noticed that what we have explored in this paper is limited to the range of tens of nanometers, methods like PNM,^91−95^ LMB,^96,97^ and machine learning^98^ could be utilized to upscale the nanometer properties to micrometer or macro scales in the future.

Based on the results of this research, possible future work could include the following: 1.Adding more functional groups or roughness to the inner surface of SWCNTs in conjunction with the tortuosity to examine their combined effects on methane transport and adsorption properties. 2.Investigating how different cross-sectional shapes, such as square, slit, or triangular, impact methane properties in tortuous nanochannels. 3.Exploring the impact of tortuosity on the adsorption and transport behavior of multiphase fluids in nanochannels.

In conclusion, the findings of this research hold significant implications for advancing our understanding of shale gas properties within shale matrices characterized by bent nanochannels. The insights gained not only contribute to the comprehension of fluid behavior at the nanoscale but also offer valuable perspectives for a broader range of applications. This includes providing insights into the behavior of other nanofluids within carbon nanotubes (CNTs) and tortuous porous media.

## Supporting Information Available

Videos of simulations trajectories correspond to cases where *r* = 5, 10, and 20 Å and *R* = 60 Å (MP4, MP4, MP4)
